# Supplementary material for: Reconciling chemical flame retardant exposure and fire risk in domestic furniture
Source: PLoS One. 2023 Nov 29;18(11):e0293651. doi: 10.1371/journal.pone.0293651 (PMC10686510; doi:10.1371/journal.pone.0293651)
Supplement: S1 File — (PDF) [file pone.0293651.s001.pdf]

Index of supplemental materials for Whaley et al. “Reconciling chemical flame retardant exposure and fire risk in domestic furniture”

Ref	Type	Description
SM01	Methods	List of Included Documents, with number of concepts identified in each
SM02	Methods	Annotation Guidelines, for document annotation process
SM03	Methods	Summary Concept Network, for fire risk
SM04	Methods	Concept Network, integrating fire risk and furniture concepts
SM05	Methods	Summary Concept Network, for CFR exposure
SM06	Methods	Concept Network, complete version for CFR exposure
SM07	Methods	Concept Network, integrating CFR exposure and furniture concepts
SM08	Methods	Concept Network, fire risk, CFR exposure and furniture integrated
SM09	Methods	Code List for fire risk and CFR exposure models
SM10	Data	Literature Analysis, for 3385 citations
SM11	Methods	Evaluation Questionnaire, fire risk, blank
SM12	Data	Evaluation Questionnaire, fire risk with volume calculations
SM13	Methods	Evaluation Questionnaire, exposure, blank
SM14	Data	Evaluation Questionnaire, exposure, with surface area calculations
SM15	Data	Data Analysis and Visualisations
SM16	Methods	Explanation of shortage of empirical data for CFR exposure model
SM17	Results	Dendrogram for fire risk clusters
SM18	Results	Scatter plot of fire risk against CFR exposure, with quadrants
SM19	Results	Sensitivity analysis
SM20	Code	Risk score calculations pseudocode
