# Supplementary material for: Reconciling chemical flame retardant exposure and fire risk in domestic furniture
Source: PLoS One. 2023 Nov 29;18(11):e0293651. doi: 10.1371/journal.pone.0293651 (PMC10686510; doi:10.1371/journal.pone.0293651)
Supplement: S3 File — (PDF) [file pone.0293651.s003.pdf]

OPSS Fire Safety: Guidelines for Annotators

Date	Comment
2021-04-30	Initial version
2021-05-12	Updated for 2 annotators
2021-05-14	Updated for 3 annotators
2021-07-13	Finalised as report appendix

About This Document **3**

Project Aims/Scope **3**

Annotation Process **4**
- Overview 4
- Workflow 4

Codes **7**
- Guidelines for Creating Codes 8

Annotation Progress **9**

Appendix A: About Atlas.ti **11**
- Documents 11
- Codes 11
- Memoes 11

Appendix B: Useful Links and References **12**

About This Document

This document contains procedures and guidance to ensure annotation of manuscripts is performed in a repeatable, standardised, manner. It is written as a set of instructions and references to aid an annotator as they examine and annotate texts.

Project Aims/Scope

Primary objective: Identify factors relating to "furniture fire safety", i.e. factors that affect the risk of a product: catching fire; spreading fire.

Secondary objective: Identify factors relating to exposure to chemical flame retardants during the use of a furniture item.

This requires coding four overall factors:

- "Involvement" i.e. characteristics of furniture related to catching fire
- "Propagation" i.e. characteristics of furniture related to spreading fire
- Exposure to flame retardants
- Vulnerability of furniture users i.e. characteristics of users that make them vulnerable to harm in the event of a furniture fire, or to exposure to flame retardants during use

The project focuses on flame retardants in current use; however, we will annotate legacy flame retardants to ensure all possible exposure information is captured. Smoke toxicity is also out of scope; however, it may impact upon furniture design features relevant to characterising fire safety. We may therefore need to annotate this.

Annotation Process

Overview

The purpose of this annotation process is to discover factors affecting fire safety within the available literature, and to enable further (automated) extraction. Coding proceeds concurrently with manuscript discovery in an iterative fashion, and this process will be supported with regular checkpoint meetings to deliver new documents.

As we are seeking to discover codes across the whole pool of literature, we're intending to do a “breadth-first” search. As such, only one instance of each code needs tagging across **all documents**. This means *if you have seen a code before, there is no need to assign it to a piece of text*. We will find all the instances of a concept later on in the project, using automated methods.

We'll deliver new literature in batches, and your input on what to include next will be factored into this. If there is a new factor in a manuscript you are annotating then record this and we'll factor it into future searches.

As this is an iterative process, we are intending to use a form of population estimation as a stopping condition. When new discoveries start slowing down, we know we're approaching the limit of what's out there, and can stop. The alternative stop condition is expert validation of the concept networks that we derive from the annotation process.

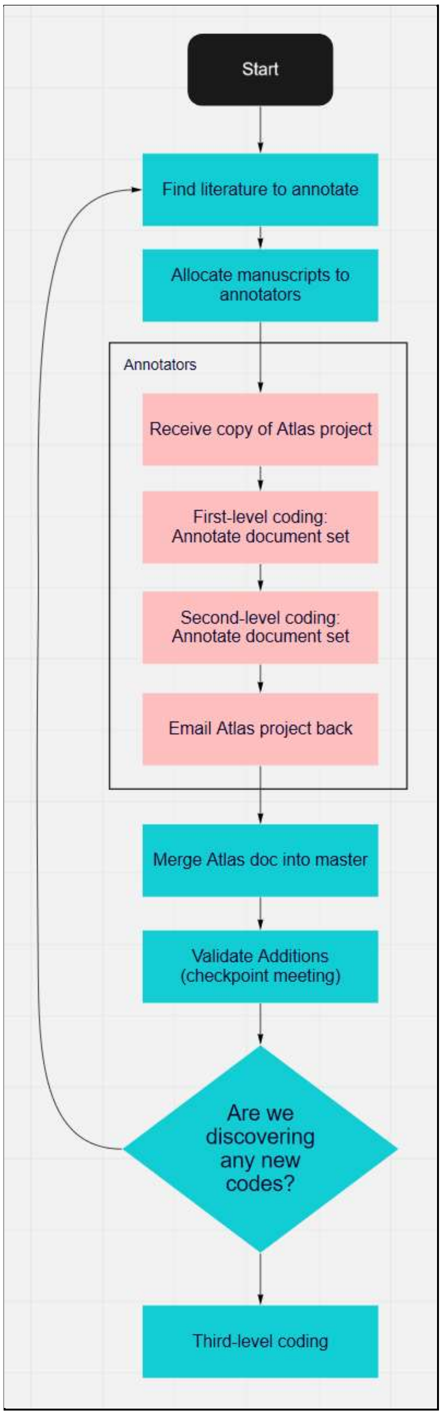

Annotation Workflow

As an annotator, you'll be provided with an Atlas.ti project containing:

- A set of numbered documents to annotate
- Code groups describing the major factors to annotate
- A memo group to suggest new literature search directions
- A memo group to record daily annotation progress (audit log)

The tagging workflow looks like this:

1. Open Atlas
2. Download the updated **Master file** for the day
3. Check the **“Annotation Progress” table** for your assigned documents
4. **For each document**:
    a. **Annotate first instance** of each factor relating to fire safety as it is mentioned in text:
        i. **Create quote** by selecting text;
        ii. **Add code** to quote by typing the text in the quote selection (click “all codes” in dialogue box. No need to assign code if the code is recognised);
        iii. **Assign code** to a fire safety factor code group (if code is new)
        iv. **Create code relationships** if you are very sure you want to commit the relationship to the project (e.g. synonyms)
    b. **On completing annotation of a document** leave a comment on the document stating “annotation complete, [your initials]”
    c. **Optional: Leave a memo** describing any new literature searches, code groups, code relationships etc. that seem like they may be useful, based on your reading of the last document. Assign the memo to the “Ideas and Suggestions” group.
5. **Write a daily memo** describing major findings of the process from the day. Assign it to the “Daily Progress Notes” memo group.
6. **At the end of the day, export** your work as a “Project Bundle” and upload it to your named folder on the shared drive
7. **Add a status note** to the “Annotation Progress” table
8. **Log your file upload** in the “File Uploads and Master File Updates” table

There is no “correct” way to code, as such, especially when it comes to assigning codes to groups. We are only trying to code in a way which makes as much sense as possible, given the constraints of consistency and what we happen to understand at the time. As this understanding will change, we will want to regularly update our annotation approach, the codes we use, and how we organise them.

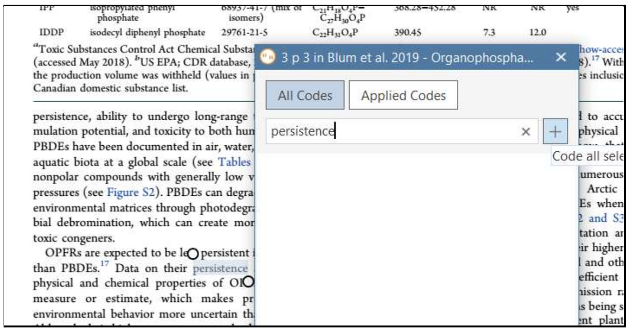

If you think a new group is needed, a code is being assigned incorrectly, that some codes are redundant, or anything else, do two things: (1) carry on coding using the agreed process, as this is essential for control and consistency in a complex iterated process, (2) create a memo for discussion on the next checkpoint call, when as a team we can decide how to change our annotation strategy based on your observation.

Also, this means you should not torture yourself with whether a code has been used already (it doesn’t matter if something is coded twice) or if you are really coding a furniture fire safety concept or if a given code really belongs to one of the code groups. The rule is: **if in doubt, code it**. We can easily eliminate codes we decide we don't want later, and it may be that marginal codes turn out to be really important.

Before each checkpoint meeting:

1. Export your Atlas project and upload it to your named Atlas Projects folder on the shared drive. The projects will get very large and therefore take time to upload on domestic internet connection. It may be necessary to plan to do this the night before a checkpoint meeting.

Codes

We are identifying concepts related to furniture fire safety and user exposure to flame retardants, i.e. concepts relating to how fires might start in furniture, be spread by furniture, the role and use of chemical flame retardants in attempting to reduce fire risks, and how people are exposed to flame retardants that originate from furnishings.

We are using a two-level coding scheme, where small terms belong to code groups thus:

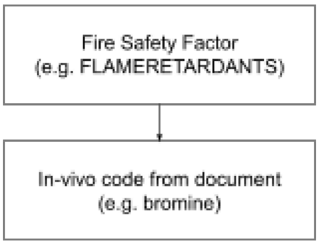

As you read a document, you should create codes as you see fit to describe the concepts therein, however, codes should then be sorted into the factors described below. As the purpose of this exercise is to *discover* codes, there is no need to code every instance of a term. Code groups are given in capital letters, starting with "grp":

Code Group	Definition
INVOLVEMENT	Catching fire
PROPAGATION	Spreading fire
FLAMERETARDANTS	Chemicals or classes of chemicals used in reducing fire risk
VULNERABILITY	User populations
EXPOSURE	Route by which populations can be exposed
FURNISHINGS	Furniture and characteristics thereof
FIRETESTS	Methods for measuring flammability and fire safety
HEALTHEFFECTS	Anything related to health effects of fire safety measures, flame retardants, smoke exposure etc.
REGULATORY	Mentions by name of specific regulatory initiatives related to flame retardants, furnishings, and furniture fire safety
SMOKE	Anything related to the production or properties of smoke, e.g. toxicity, visibility, generation etc.

zTODISCUSS	Any code which is not easily classified that we should talk about
zFORDELETION	For any codes that need to be deleted from the Master file
zTRAINING	For any codes that need to be communicated to the group for modifying or clarifying the coding strategy

In addition to the larger groups, we suggest using code relations to link codes that are very obviously similar, e.g. "tetrabromobisphenol a" and "TBBPA", by dragging one on top of another and using a "is synonym" relation. There are some other relations that can be used too, and we may add to this list following discussion at checkpoint meetings:

**Relation Type (green included, others excluded)**	**Usage**
contradicts	N/A
**is a**	Used where one concept is entirely subordinate to another, i.e. "TBBPA" *is a* "flame retardant"
**is a property of**	When one concept is a property of another, e.g. “volume is a property of cushion”
is associated with	Bidirectional association, use sparingly
is cause of	When one concept is stated to cause another concept. Does not need to be true, just claimed.
**is part of**	When one concept is part of another, e.g. “HCN is part of smoke”, “chair leg is part of chair”
is synonym of	Used to link initialisms and codes that only vary due to small typographical choices, i.e. "tetrabromobisphenol" and "tetrabromobisphenol-a"
**affects**	Anything that affects some kind of outcome

Annotation Progress

At the start of each day, check this table to see which documents you have been assigned. Some will be reassigned depending on annotator progress (e.g. some documents are very long so will take one annotator a long time to do).

This document is live, so indicate when you have started annotation by typing “started” into the status column, and when you have finished by typing “completed”.

When you have finished annotating a document, don’t forget to add a comment to the document with your initials stating the annotation is complete.

Document Number	Designated Annotator	Status + Date (started / completed)
2		Completed / 17:11 on 14 May
3		Completed
4		Completed 18 may
5		Started 19 may completed
6		Started 19 may and completed
7		Started up to P9/31, 21 May., cpmpleted
8		complete
9		Completed 25 May
10		Completed 25 May
11		Completed 21 May
12		Completed 18 May
13		Completed 19 May
14		Completed 23 May
15		Completed
16		Completed
17		Completed 19.05.21
18		Completed 19.05.21
19		Started 19.05.21, completed 15th June
20		Started 24 may, completed

21	[redacted]	Started 21 May, completed
22	[redacted]	Completed 23/5/21
23	[redacted]	Completed 13/7/21
24	[redacted]	Completed 09/06/21
25	[redacted]	Completed 09/06/21
26	[redacted]	Annotate Exec Summary only completed 1/6/21
27	[redacted]	Completed 3/6/21
28	[redacted]	Completed 6/6/21
29	[redacted]	Completed 2/6/21
30	[redacted]	Annotate report body only, not appendices. Completed 3/6/21
31	[redacted]	Completed 13/7/21
32	[redacted]	Completed 10/6/21

Network mapping

Code group (green = critical, grey less important, yellow = supplementary)	Who did it?	Status and date
~~grp_ENVIRONMENTALBEHAVIOUR~~	[redacted]	~~Less important~~
**net_EXPOSURE**<br>Terminus: HUMAN EXPOSURE PATHWAY	[redacted]	Critical, currently **v2 done**
~~**grp_FIRETESTS**~~	[redacted]	~~Not actually very helpful for task, except for providing a burn orientation concept~~
~~grp_FLAMERETARDANTS~~	[redacted]	~~Hold off on this one for now, looks complicated~~
**net_FURNISHINGS**<br>Terminus: SMALL UPHOLSTERED FURNISHINGS	[redacted]	Critical, currently **v2 done**
~~grp_HEALTHEFFECTS~~	[redacted]	~~Hold off~~
**net_INVOLVEMENT_OBJECT**<br>Terminus: IGNITABILITY<br><br>**net_INVOLVEMENT_SOURCE**<br>Terminus: IGNITION SOURCE	[redacted]	Critical, **after INVOLVEMENT was revised on RH input, split into ignitability of object and source of ignition, currently v2 done**
**grp_PROPAGATION**<br>Terminus: FLAME SPREAD	[redacted]	Critical, currently **v3 done [updated for concepts that apply to burning of individual items of**

		**furniture, much happier with it now]**
~~grp_REGULATIONS~~	[redacted]	~~Hold off, does not look especially useful~~
~~grp_SMOKE~~	[redacted]	~~Hold off, out of scope~~
**grp_VULNERABILITY**<br>Terminus: VULNERABILITY	[redacted]	Critical, **v1 done**
ISO VOCAB	[redacted]	Not strictly a group, but can check for concepts missing from networks. **Done**.
	[redacted]	

File Uploads and Master File Updates

The table below is to help us keep track of when an annotator has uploaded an updated file. The date and time when a new master file is made available will also be logged.

- Annotators should **upload** a copy of their file to their folder on the shared drive at the **end of each working day**.

Note: The **process for creating a new Master file** is as follows:

1. Annotators will be asked to **submit** their current file on a specified deadline
2. The annotators will then **cease work** until they are passed the updated Master file
3. PW will create a new master file by merging the current files
4. There will be a check-in meeting to discuss progress and issues
5. After the check-in meeting, the new master file will be distributed and annotation work can continue.

Annotator Initials	Date and Time of Update	Type of update	Summary of changes and other notes
[redacted]	6pm 13/5/21	Uploaded project bundle to my folder	Partial completion of doc 3, some changes to code associations
[redacted]	17:11 14/5/21	Uploaded project bundle to my folder	Completed doc 3
[redacted]	14:19 18/5/21	[redacted]	[redacted]
[redacted]	19/05/21 16:25	[redacted]	[redacted]
[redacted]	20/5/21	[redacted]	[redacted]

[redacted]		Housekeeping: 3, 4, 5, 6, 7	
[redacted]	21/05/21	Uploaded project bundle to my folder	Updated docs 21 and 7
[redacted]	21/05/25	Uploaded project bundle to my folder	All docs completed
[redacted]	27/5/21	[redacted]	[redacted]
[redacted]	3/6/21	[redacted]	[redacted]
[redacted]	4/6/21	[redacted]	[redacted]
[redacted]	09/06/21	[redacted]	[redacted]
[redacted]	9/6/21	[redacted]	[redacted]
[redacted]	16/6/21	[redacted]	[redacted]
[redacted]	12/7/21	[redacted]	[redacted]

Guidelines for merging projects

1. Import each project and check for changes
2. When satisfied, merge
3. Clean up code list
4. Add or modify code groups
5. Add new documents and document groups
6. Add a memo with information for the team
7. Save as Master
8. Distribute

Guidelines for concept mapping

Colour codes[1]

Note that not all of these colour codes will be present in a final network; these are used and changed throughout coding as a way for the coders to annotate their current use.

Colour	Meaning	Notes
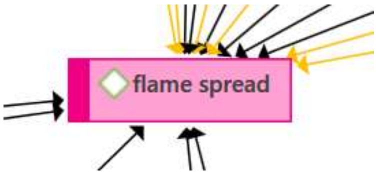	Node for thinking about more	
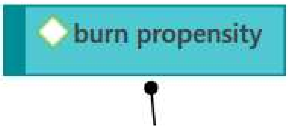	Questionable value in network	Might be falsely related, misallocated to a network, not useful, artifact of the network rather than fire safety, etc..
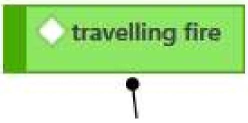	Free code added from expert input	Code not found in the annotated documents but suggested by an expert (e.g. to make sense of other code relationships, add a missing concept, etc.)
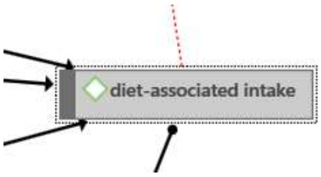	Terminus, excluded	To show a node that is a network terminus that is excluded from the model (for transparency)
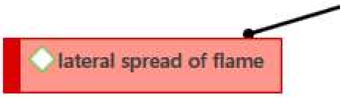	Node highlighted for further investigation	
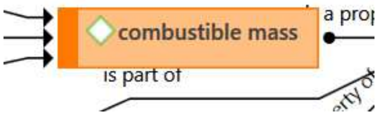	Prospective key node	

[1] Note that these colours were used to facilitate the mapping and concept analysis process; the final colours in the matrix are different and represent the role of a concept in the matrix model.

Appendix A: About Atlas.ti

Atlas.ti is a leading piece of CAQDAS software, offering facilities for document annotation and analysis of the resulting annotations. Here we are using three main facilities:

Documents

Each document represents a single manuscript in this study. Documents can be assembled into document groups, which can overlap (that is, a document may be in more than one group).

Codes

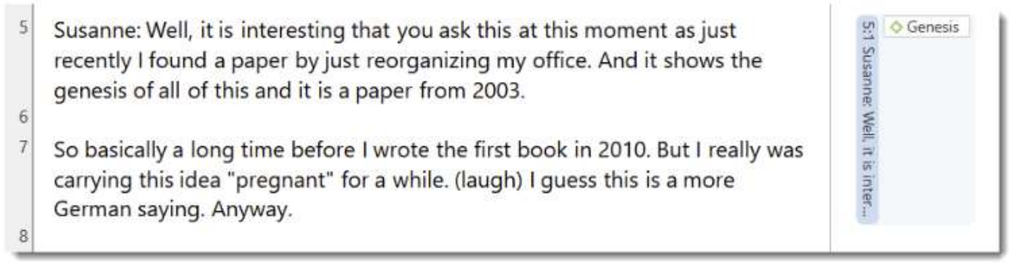

Codes in Atlas are small tags that can be assigned to part of a document.

Codes can be created and managed without annotating a document (free coding), or assigned during the annotation process. Creating a code 'from' the text is referred to in Atlas as coding "in-vivo". Codes are assigned to *quotes*, which describe a section of document.

Codes can also be assigned to groups, and we have populated the Atlas.ti project with groups representing cases we are interested in.

Coding	Short-Cut
Create Free Code	Ctrl+K
Apply Codes	Ctrl+J
Quick Coding	Ctrl+L
Code In-Vivo	Shift + Ctrl + V

Memos

Memos are simply text documents to record information. We use them on this project as a space to note down anything that doesn't fit cleanly into annotations.

Appendix B: Useful Links and References

- Annotators' supporting documents on Google Drive
- Atlas.ti download link: windows, mac
- Atlas.ti documentation
