# Supplementary figures and images for: Reconciling chemical flame retardant exposure and fire risk in domestic furniture
Source: PLoS One. 2023 Nov 29;18(11):e0293651. doi: 10.1371/journal.pone.0293651 (PMC10686510; doi:10.1371/journal.pone.0293651)

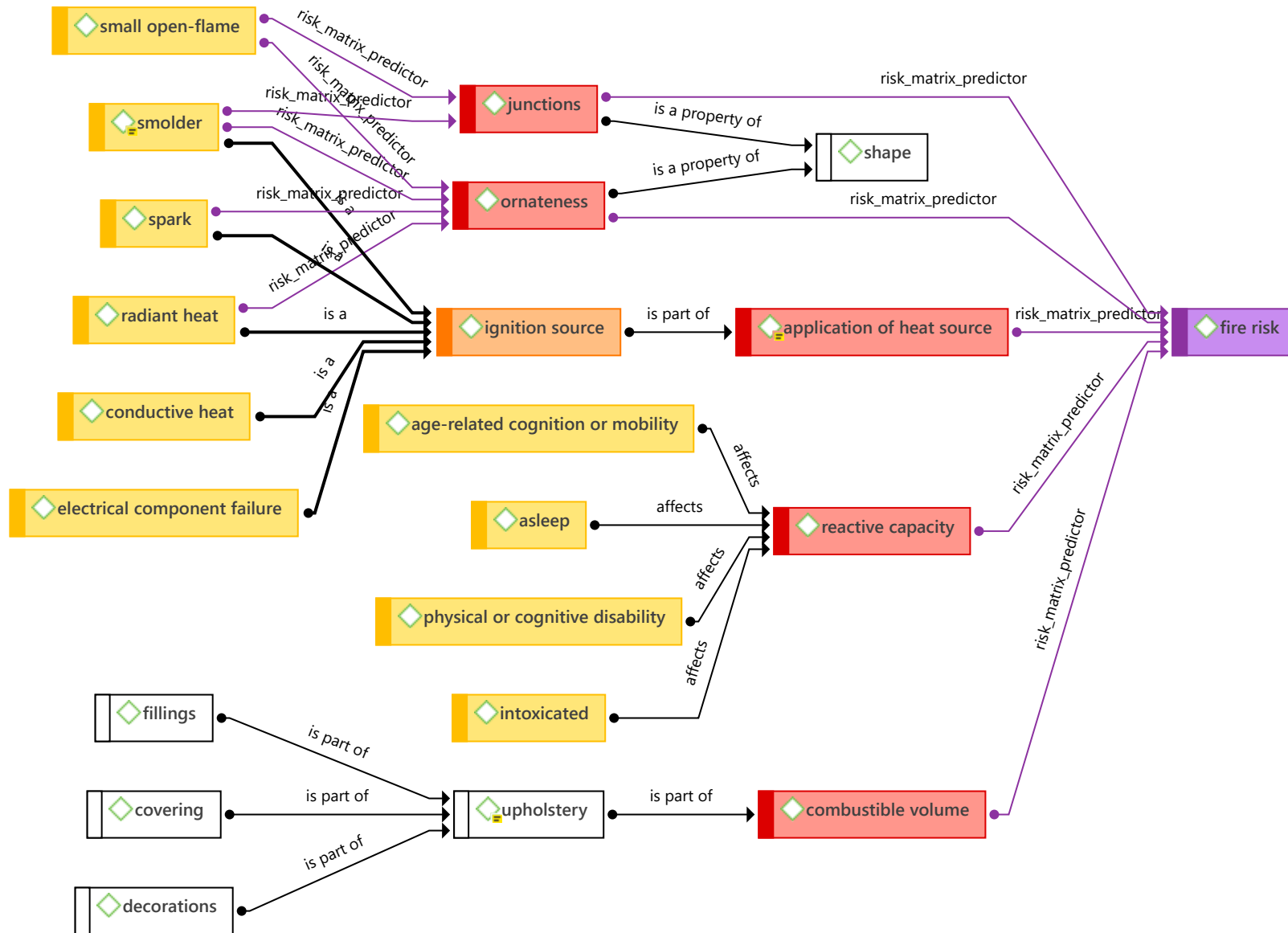

Supplement: S4 File — (PDF) [file pone.0293651.s004.pdf]

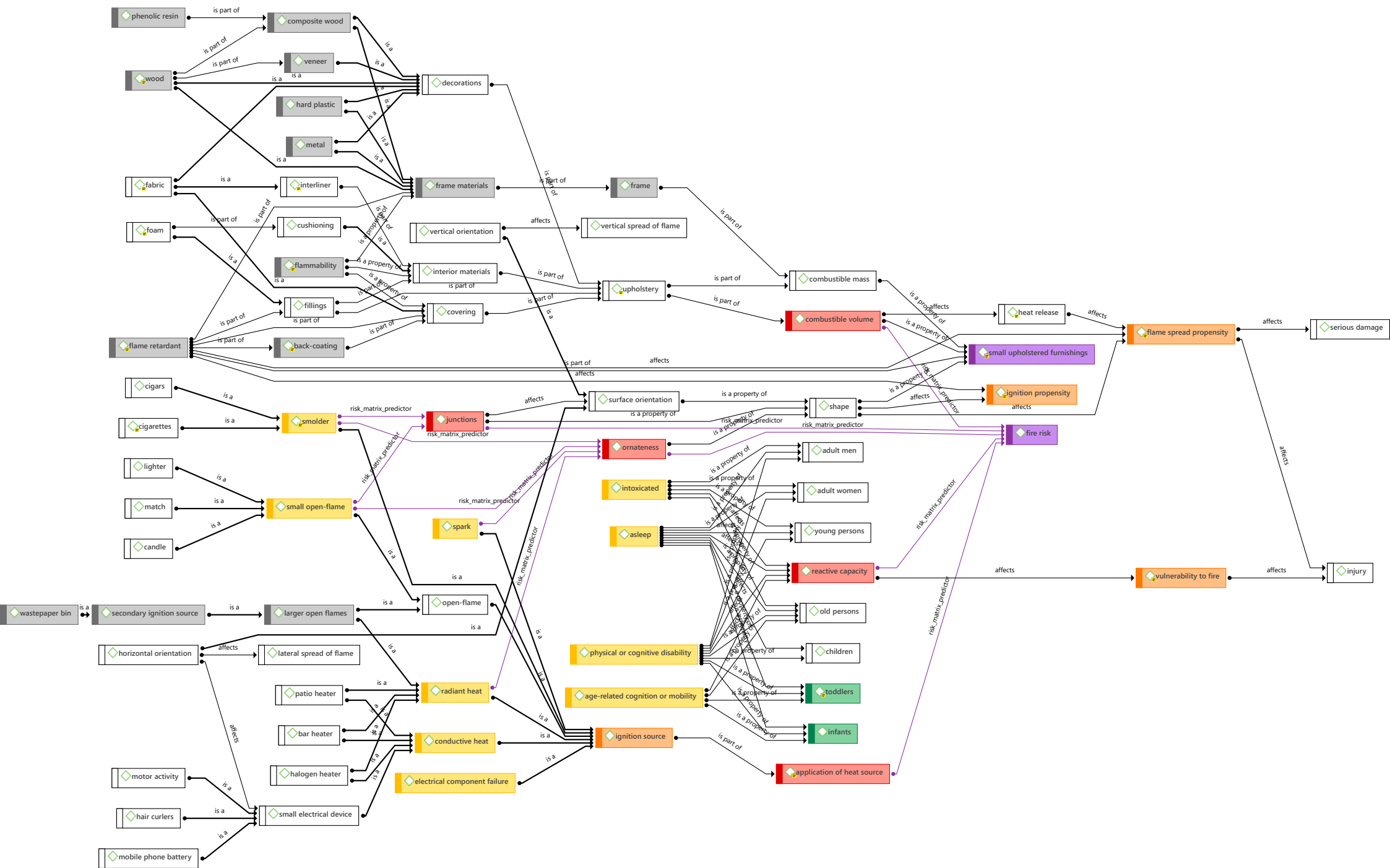

Supplement: S5 File — (PDF) [file pone.0293651.s005.pdf]

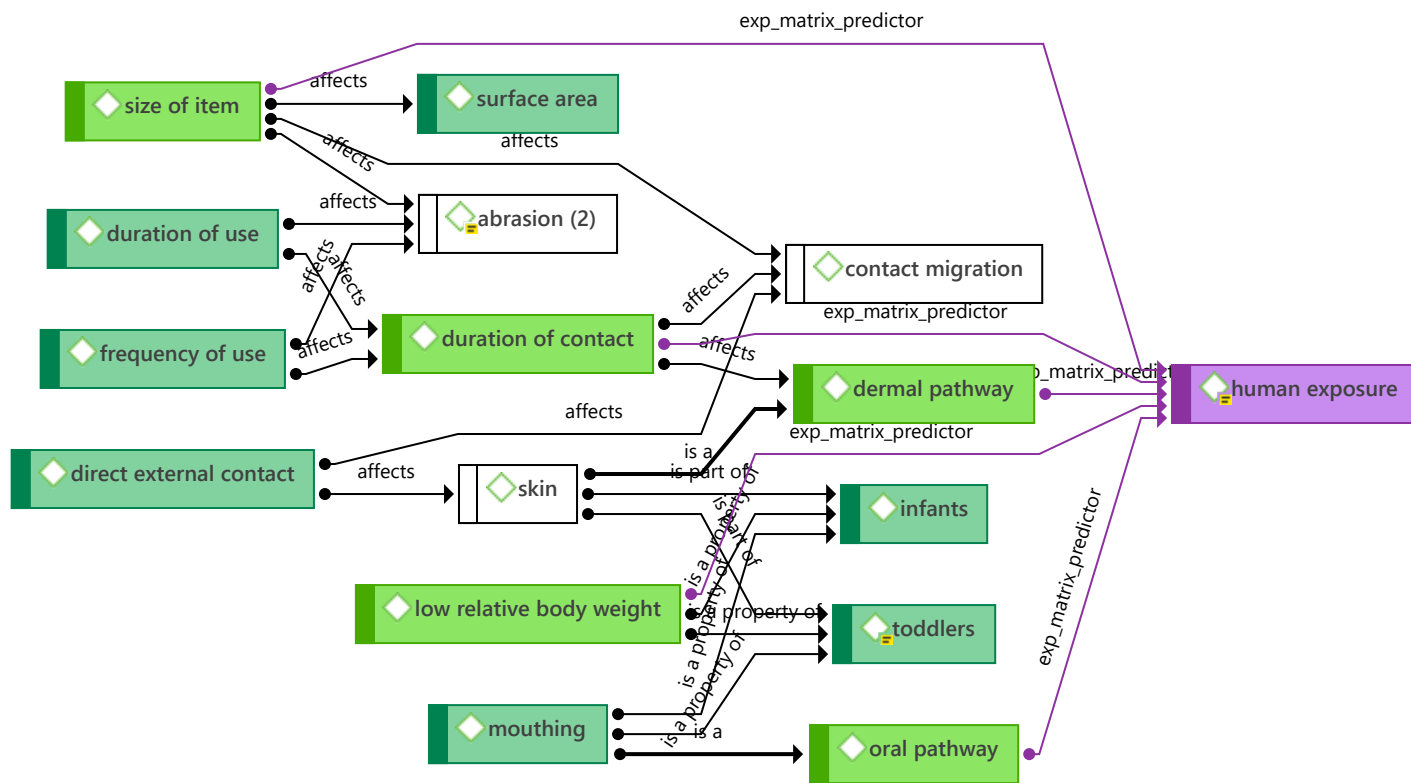

Supplement: S6 File — (PDF) [file pone.0293651.s006.pdf]

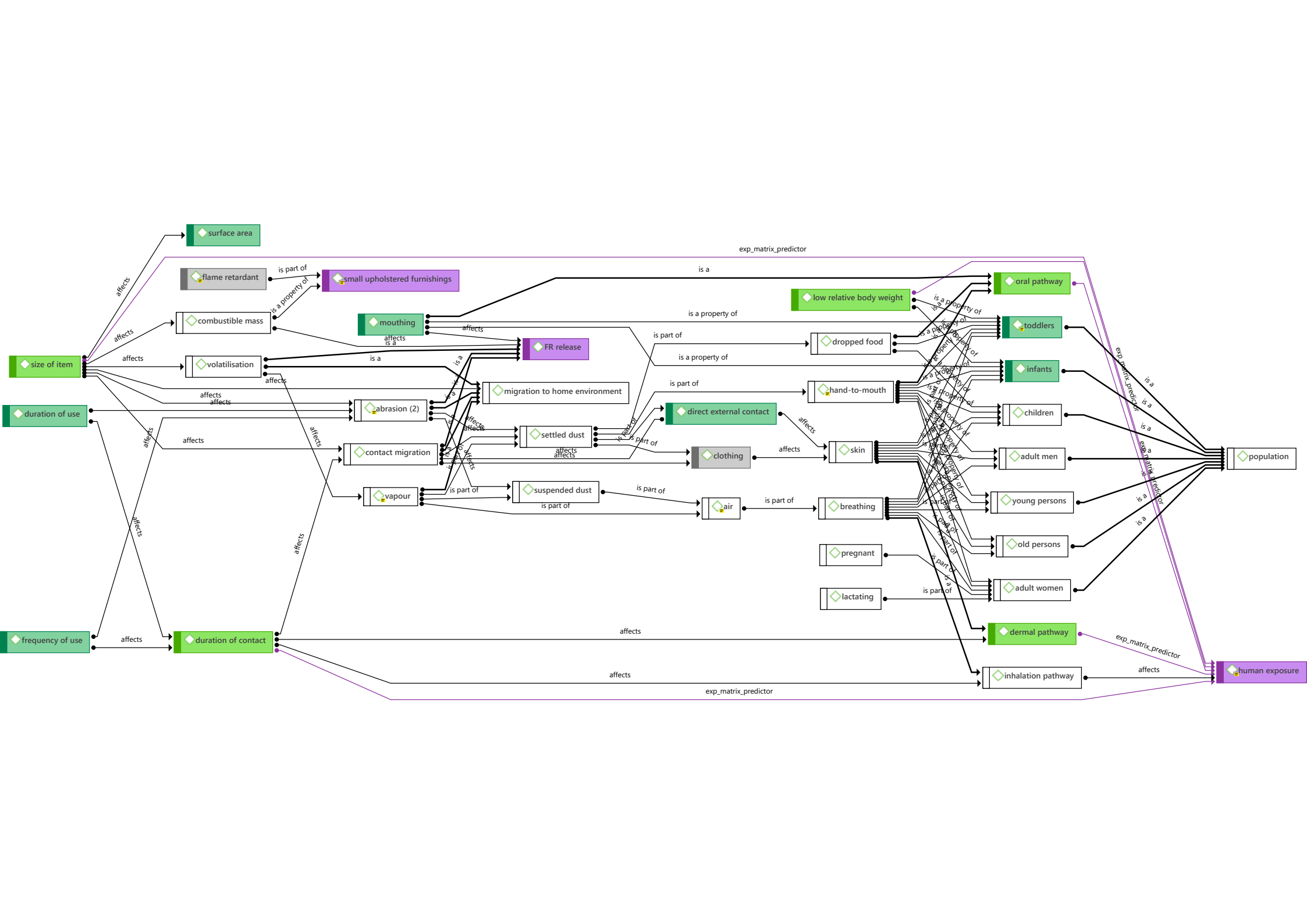

Supplement: S7 File — (PDF) [file pone.0293651.s007.pdf]

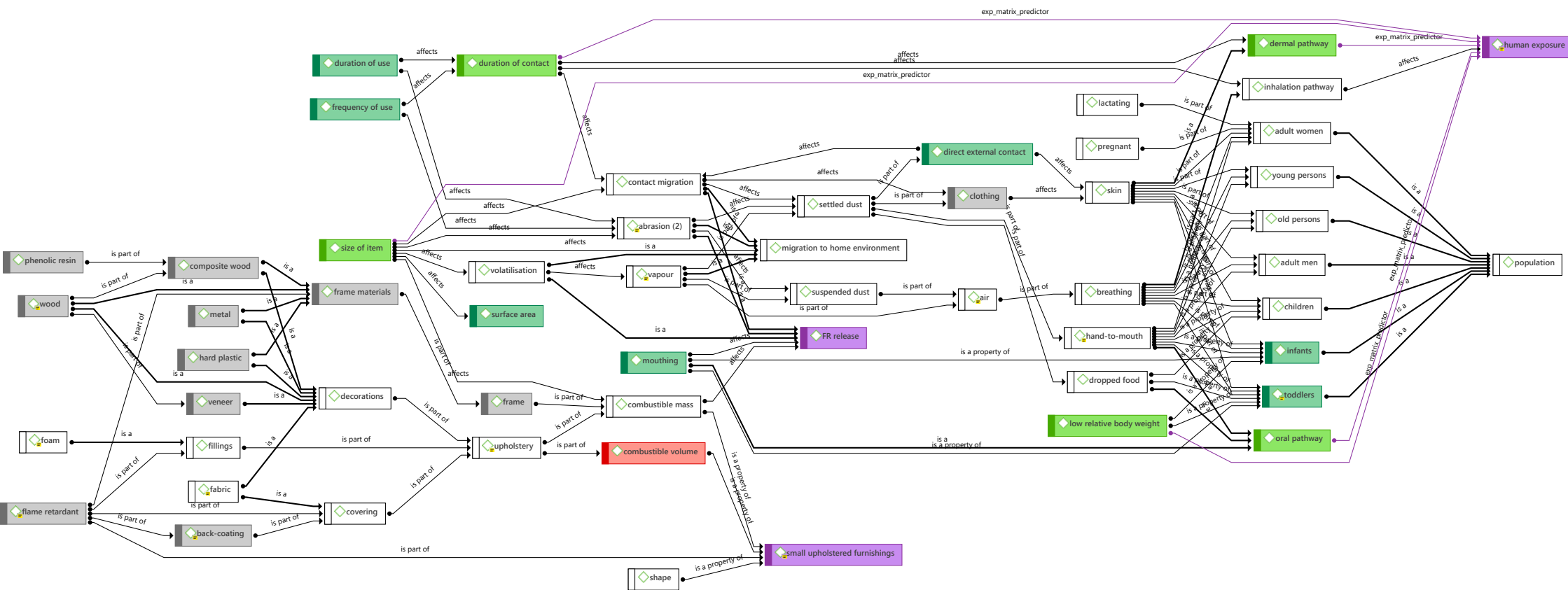

Supplement: S8 File — (PDF) [file pone.0293651.s008.pdf]

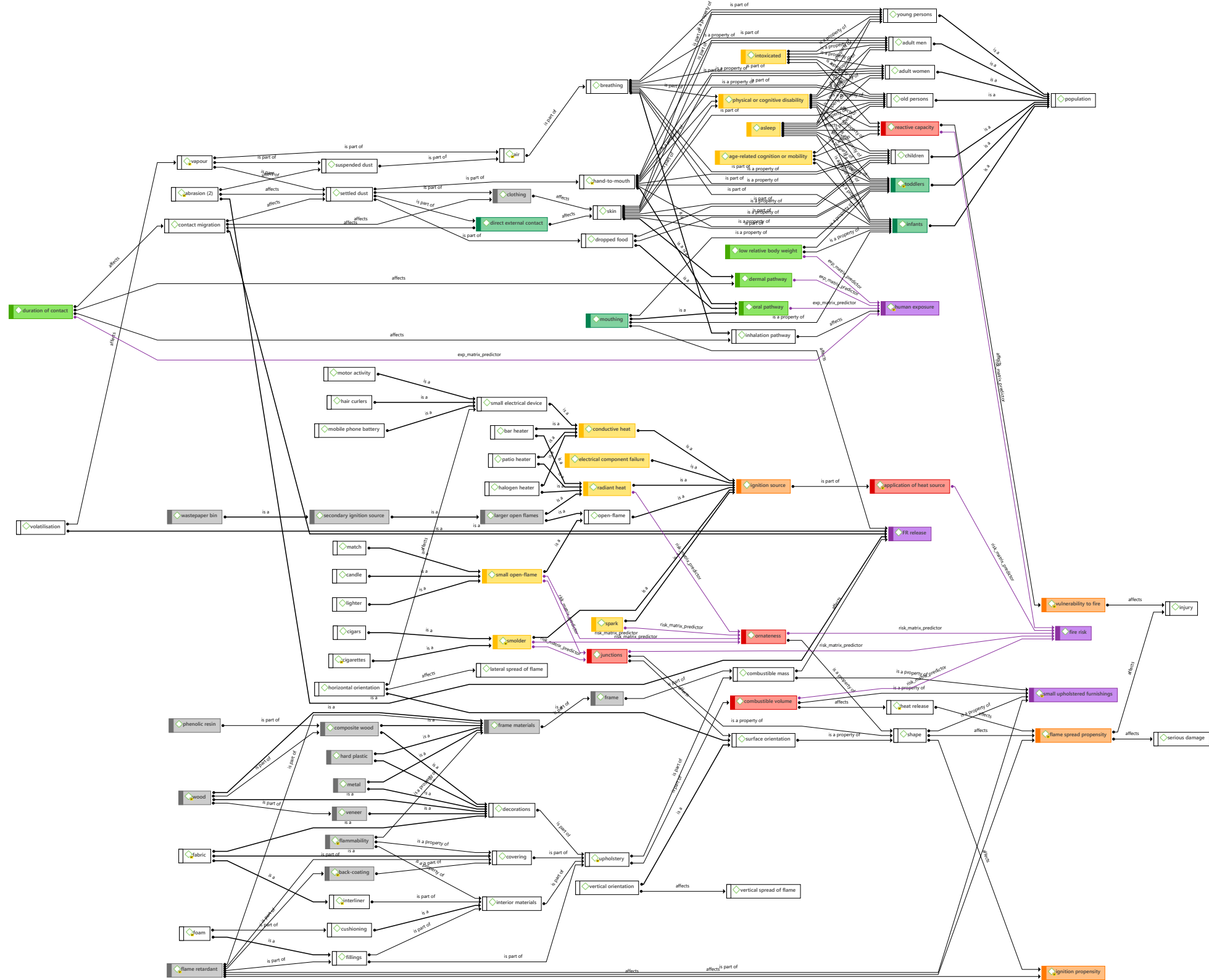

Supplement: S9 File — (PDF) [file pone.0293651.s009.pdf]

Similarity Dendrogram (Injury, Damage, Exposure; Euclidean Distance)

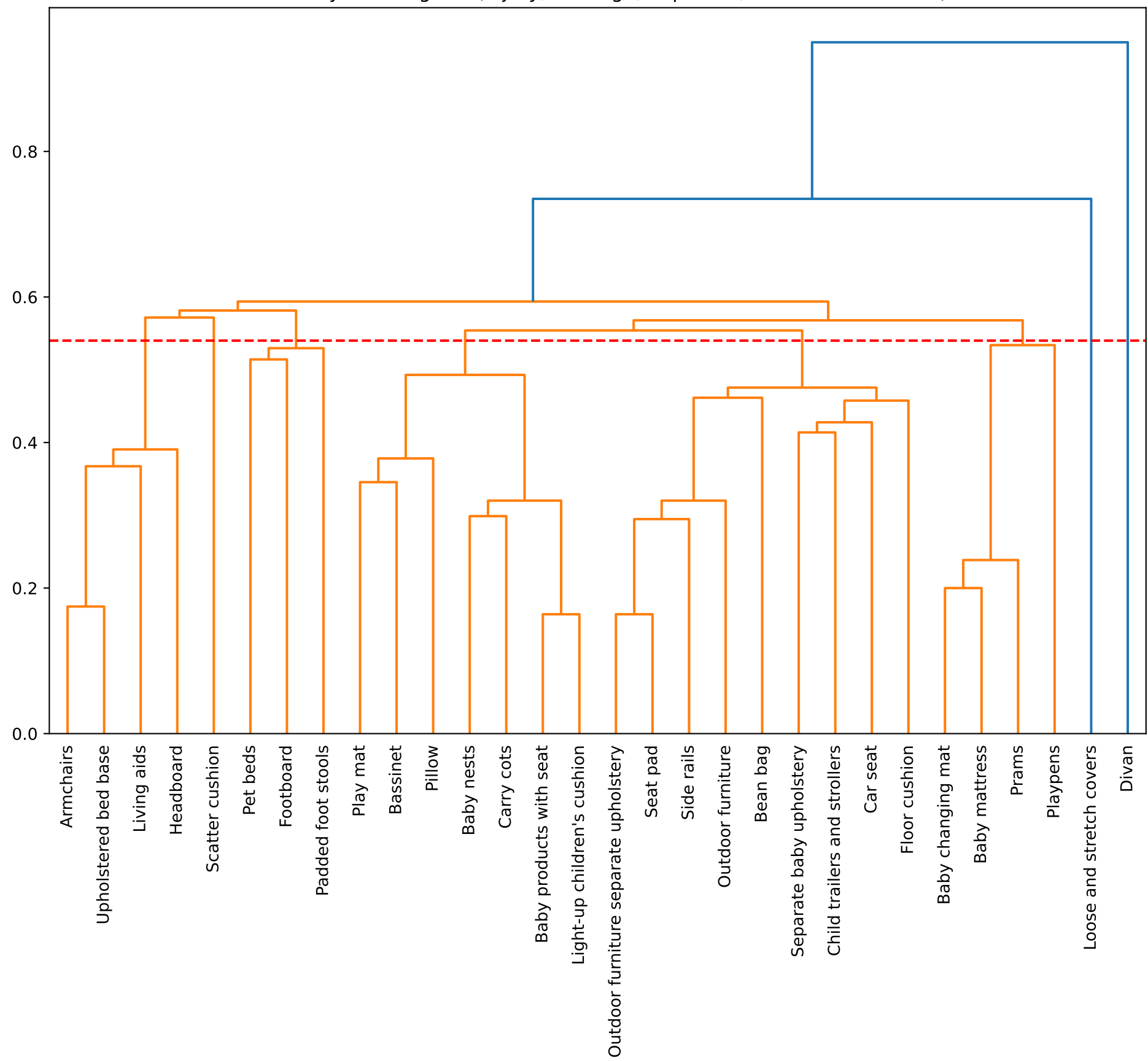

Supplement: S18 File — (PDF) [file pone.0293651.s018.pdf]

Exposure vs. Injury Risk (low/high shaded)

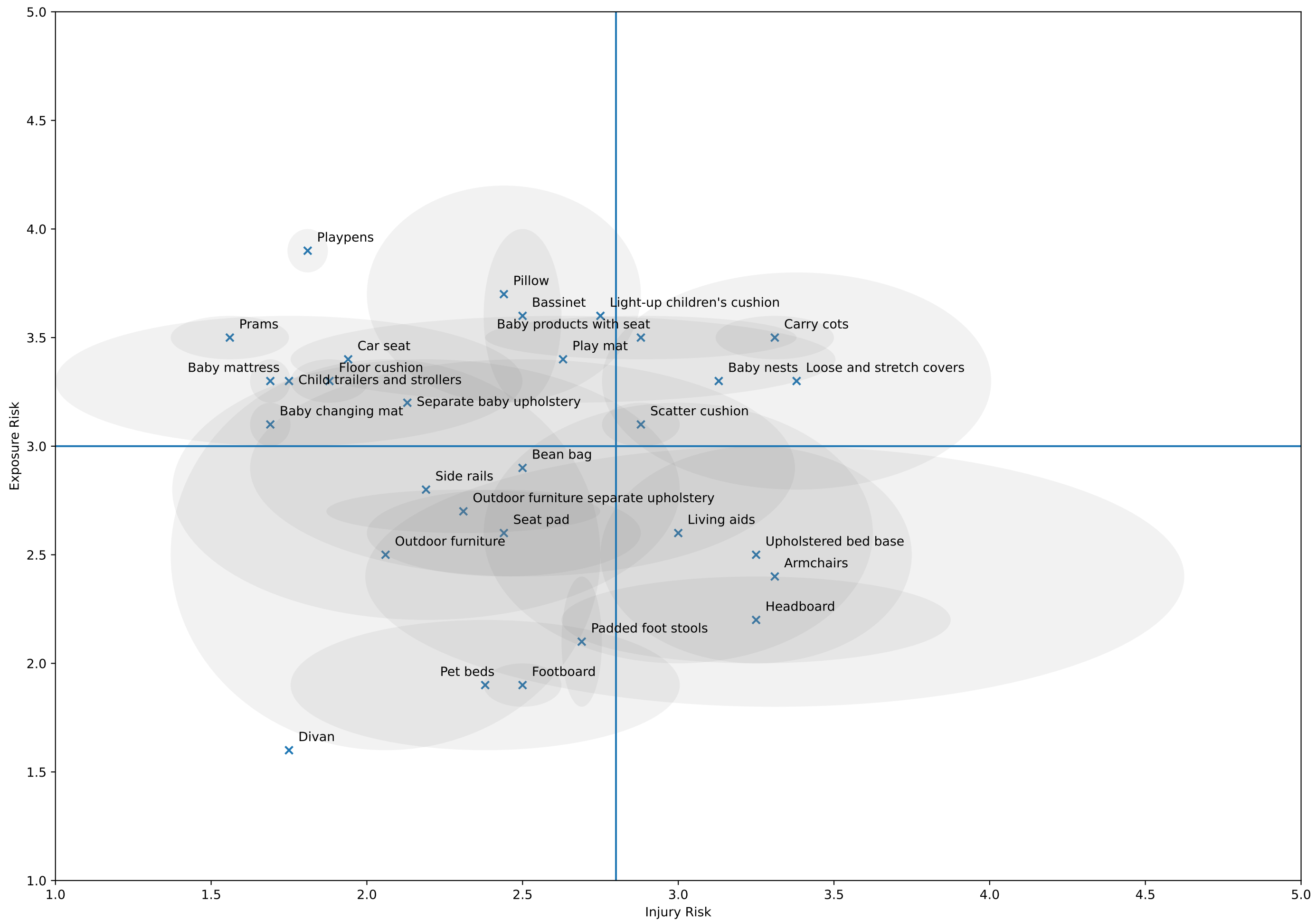

Supplement: S19 File — (PDF) [file pone.0293651.s019.pdf]
