# Supplementary material for: Reconciling chemical flame retardant exposure and fire risk in domestic furniture
Source: PLoS One. 2023 Nov 29;18(11):e0293651. doi: 10.1371/journal.pone.0293651 (PMC10686510; doi:10.1371/journal.pone.0293651)
Supplement: S17 File — (DOCX) [file pone.0293651.s017.docx]

**Responses to questions related to exposure to CFRs used in furniture**

- *Does FR usage vary between furniture categories?*

The FR of choice may differ between applications depending on other factors such as: regulatory standard required to be met and FR stability or other properties (such as “fogging”) under conditions experienced in a given application. Overall, it is likely that a variety of FRs may be used across a different furniture category. To illustrate, in 22 samples of waste furniture fabrics collected from waste sites in the Republic of Ireland in 2015-16, hexabromocyclododecane (HBCDD) was present at concentrations between 2.1 % and 5.1 % in 6 samples; with decabromodiphenyl ether detected at between 0.3 % and 7.3 % in 6 samples (Harrad et al, 2019). In the UK, 7 out of 9 waste domestic and office furniture foam samples collected in 2011-12, were treated with tris(2-chloroisopropyl)phosphate (TCIPP) at concentrations ranging from 0.8 % to 3.8 %; with another single foam sample containing tris(1,3-dichloroisopropyl)phosphate (TDCIPP) and tris(2-chloroethyl)phosphate (TCEP) at 1.1 and 0.5% respectively (Stubbings et al, 2016).

- *What proportion of overall exposure to FRs derives from their use in furniture compared to other applications such as EEE?*

In brief, the answer is that this is very hard to quantify accurately. At a very simple level, where application of a specific FR is essentially limited to domestic furniture only, then one can assume all exposure may be attributed to this application. In other scenarios, it could be assumed to reflect information on the proportion of a given FR that is used in domestic furniture compared to other applications. Such a simple approach may have some validity for TDCIPP. This is because the EU RAR for TDCIPP notes that most TDCIPP is used in the production of flexible polyurethane (PUR) foam (EU, 2008a). The EU RAR further notes that most foams containing TDCIPP are used in the automotive industry in applications like seat cushions, with some use in furniture, with the latter accounting for ~15-20 % of total EU use. As the pathways of emission and exposure to TDCIPP will be broadly similar between both these vehicular and domestic applications, it is not unreasonable to assume that ~15-20 % of population-level exposure to TDCIPP is attributable to its use in furniture foam. However, this is not the case for all FRs, as the extent of exposure that arises may depend substantially on the application, in a fashion that is hard to quantify accurately without further research. As an illustration, the EU risk assessment report for TCIPP records that the majority (80%) of TCIPP applied in the EU was used in rigid PUR foam for construction applications, with flexible PUR foam in upholstery and bedding accounting for a further 17% (EU, 2008b). It should be noted that this latter figure will likely be greater in the UK, as the EU RAR notes that TCIPP use in flexible foam for furniture was for the UK and Irish market. However, it is likely that exposure arising from the application of TCIPP in construction foam (such as that used for building insulation) will be restricted largely to inhalation of vapour and suspended dust, as well as ingestion of and dermal uptake from settled surface dust. All such exposure pathways will arise following volatilisation of TCIPP from building insulation foam. The extent to which such volatilisation impacts on concentrations in indoor air and dust, will likely be lower where TCIPP is used in insulation foam fitted in cavity walls where the wall will to some extent sorb any volatilised FR, than if it were used in furniture foam, where volatilisation to air will occur relatively unimpeded. Moreover, emissions via pathways such as abrasion of furniture fibres contaminating indoor air and dust, as well as transfer to dust settled on furniture surfaces will not occur when TCIPP is used in cavity wall insulation foam. Furthermore, substantial exposures to TCIPP used in furniture can occur via mouthing of the treated item, as well as dermal uptake. Neither of these exposure pathways are likely when TCIPP is used in insulation foam. In summary, the likely contribution of furniture to overall exposure to TCIPP is likely to exceed the proportion of it used in furniture as opposed to other applications, but there are too many uncertainties at this time for this to be quantified reliably.

- *How does the proportion of overall exposure to FRs deriving from use in furniture compared to other applications vary between exposure groups (e.g. children, adults, elderly…)?*

With respect to near-field indoor exposure, young children likely practise mouthing behaviour to a far greater extent than older children and adults. As mouthing behaviour is likely a far greater exposure pathway for FRs present in furniture than in other applications such as construction foam and electrical and electronic equipment (EEE), young children are likely more exposed to FRs in furniture than adults. Moreover, adults likely have greater contact with FRs in EEE, thereby reducing the proportion of their exposure attributable to furniture. Overall, it is thus likely that a greater proportion of young children’s exposure to FRs stems from furniture, than for adults. However, data are not available that would allow this to be quantified accurately.

**References**

- Stuart Harrad, Daniel Drage, Mohamed Abdallah, Martin Sharkey and Harald Berresheim (2019). Evaluation of Hand-held XRF for Screening Waste Articles for Exceedances of Limit Values for Brominated Flame Retardants. EPA of Ireland Research Report No. 272. ISBN: 978-1-84095-820-1 available at <https://www.epa.ie/publications/research/waste/research-272-evaluation-of-hand-held-xrf-for-screening-waste-articles-for-exceedances-of-limit-values-for-brominated-flame-retardants.php>
- William A. Stubbings, Daniel S. Drage, Stuart Harrad (2016). Chlorinated organophosphate and “legacy” brominated flame retardants in UK waste soft furnishings: A preliminary study. *Emerging Contaminants* 2, 185-190.
- EU RAR (2008a). European Union Risk Assessment Report TRIS[2-CHLORO-1-(CHLOROMETHYL)ETHYL] PHOSPHATE (TDCP)
- EU RAR (2008b). European Union Risk Assessment Report TRIS(2-CHLORO-1-METHYLETHYL) PHOSPHATE (TCPP)
