# Supplementary material for: Reconciling chemical flame retardant exposure and fire risk in domestic furniture
Source: PLoS One. 2023 Nov 29;18(11):e0293651. doi: 10.1371/journal.pone.0293651 (PMC10686510; doi:10.1371/journal.pone.0293651)
Supplement: S21 File — (DOCX) [file pone.0293651.s021.docx]

| # Lookup tables, used to normalise observed scores into a 1-5 range. JUNCTION_RISK_TABLE = [0.0, 1.0, 2.0, 3.0, 4.0] COMBUST_RISK_TABLE = [0, 0.018, 0.036, 0.072, 0.09] LOG_SURFACE_AREA_TABLE = [-2.407945609, -1.131492912, 0.1449597837,  1.42141248, 2.697865176]  # Used to return a score 1-5 based on the min values in the table given def lookup(raw: float, table: list[float]) -> int:  for i, minval in enumerate(table):  if minval > raw:  return i   return 5  # Return an injury risk score, 1-5 inclusive def injury_risk(exposure_to_smokers: bool, contact: int, ornateness: int,  combust_volume: float, junctions: int, reactivity: int):   # Exposure to Ignition Source  exposure = max(1, min(contact, 5))   # Ignition of item  ignition = lookup(junctions, JUNCTION_RISK_TABLE) if exposure_to_smokers else 0  ignition = (ignition + ornateness) / 2   # Flame Spread  flame_spread = lookup(combust_volume, COMBUST_RISK_TABLE)   # Matrix 'vulnerability' is simply the reactivity score.  scores = [exposure, ignition, flame_spread, reactivity]  return quantile(list(scores), 0.25)  # Return a damage risk score, 1-5 inclusive def damage_risk(exposure_to_smokers: bool, contact: int, ornateness: int,  combust_volume: float, junctions: int):   # Exposure to Ignition Source  exposure = max(1, min(contact, 5))   # Ignition of item  ignition = lookup(junctions, JUNCTION_RISK_TABLE) if exposure_to_smokers else 0  ignition = (ignition + ornateness) / 2   # Flame Spread  flame_spread = lookup(combust_volume, COMBUST_RISK_TABLE)   scores = [exposure, ignition, flame_spread]  return quantile(list(scores), 0.25)  # Return an exposure risk score, 1-5 inclusive def exposure_risk(low_rel_body_weight: int, bare_skin: int, mouthing: int,  duration: int, surface_area: float):   normalised_surface_area = lookup(log(surface_area),  LOG_SURFACE_AREA_TABLE)  scores = [low_rel_body_weight, bare_skin, mouthing,  duration, normalised_surface_area]   return sum(scores) / len(scores) |
| --- |
