# Supplementary material for: Reconciling chemical flame retardant exposure and fire risk in domestic furniture
Source: PLoS One. 2023 Nov 29;18(11):e0293651. doi: 10.1371/journal.pone.0293651 (PMC10686510; doi:10.1371/journal.pone.0293651)
Supplement: Attachment — Submitted filename: Response to reviewer comments - PLoS Whaley et al.docx [file pone.0293651.s022.docx]

Response to reviewer comments

**Whaley et al. “Reconciling chemical flame retardant exposure and fire risk in domestic furniture”**

We thank the handling editor and reviewers for their comments and suggestions for this manuscript.

We have provided the additional information requested by the handling editor. With regard to our funding disclosure and declaration of competing interests, we have updated the declarations and provided a specific financial disclosure as requested. In conjunction with the statement of author contributions, we believe the information about funding and funder role are comprehensive and accurate, but have requested guidance as to any further information that we can provide.

Review 1 requested additional context. We have provided information about regional differences in approach to regulation of CFRs via comment and citation of the suggested references. To address Reviewer 1’s request for additional discussion of differing risk profiles of subgroups of flame retardant, change in use of particular substances over time, and relative incidence of fires and deaths, we have provided a link to the government report related to this study, where these issues are discussed in appropriate detail, and clearly signposted that this is where a reader can access more of this contextual information (see responses below).

All other comments have been addressed.

We thank the editors and reviewers once again for the time and energy they have put into commenting on our manuscript.

With best wishes, on behalf of the author team,

Paul Whaley (21 September 2023)

| Ref | Comment | Response |
| --- | --- | --- |
| Journal requirements / comments from Handling Editor | | |
| 1 | 1. Please ensure that your manuscript meets PLOS ONE's style requirements, including those for file naming.  The PLOS ONE style templates can be found at  <https://journals.plos.org/plosone/s/file?id=wjVg/PLOSOne_formatting_sample_main_body.pdf> and  <https://journals.plos.org/plosone/s/file?id=ba62/PLOSOne_formatting_sample_title_authors_affiliations.pdf> | We have updated the typesetting and citation format to comply with the referenced style guides.  We have saved the images in TIF format but the resolution for several of these may be low for print. We have also created SVG files where appropriate (we do not have access to Adobe software for EPS file creation but this format should work). |
| 2 | 2. Thank you for stating the following financial disclosure:  [The research was funded under competitive tender "CR21021 - Fire Risks of Upholstered Products" as part of the UK Department for Department for Business, Energy & Industrial Strategy (BEIS) Office for Product Safety and Standards (OPSS) Strategic Research Programme. The contract was awarded to University of Central Lancashire with TRH as Principal Investigator. PW, SW, NB, SH, and EP were engaged as subcontractors. TK and DN were involved as BEIS employees in study design and preparation of the manuscript. The manuscript went through an internal clearance process at BEIS and OPSS before being approved for submission.]  Please state what role the funders took in the study. If the funders had no role, please state: ""The funders had no role in study design, data collection and analysis, decision to publish, or preparation of the manuscript.""  If this statement is not correct you must amend it as needed.  Please include this amended Role of Funder statement in your cover letter; we will change the online submission form on your behalf. | We have provided in the cover letter the following text about the role of the funder (see also comment 5 below):  The research was funded under competitive tender "CR21021 - Fire Risks of Upholstered Products" as part of the UK Department for Department for Business, Energy & Industrial Strategy (BEIS) Office for Product Safety and Standards (OPSS) Strategic Research Programme. Two BEIS employees (TK, DN) were involved in study design and preparation of the manuscript. The manuscript went through an internal clearance process at BEIS and OPSS before being approved for submission. The specific roles of each author in the study are articulated in the “Author contributions” section of this manuscript. |
| 3 | 3. We note that Figures 3 and 4 in your submission contain copyrighted images. All PLOS content is published under the Creative Commons Attribution License (CC BY 4.0), which means that the manuscript, images, and Supporting Information files will be freely available online, and any third party is permitted to access, download, copy, distribute, and use these materials in any way, even commercially, with proper attribution. For more information, see our copyright guidelines: <http://journals.plos.org/plosone/s/licenses-and-copyright>.  We require you to either (1) present written permission from the copyright holder to publish these figures specifically under the CC BY 4.0 license, or (2) remove the figures from your submission:    1.) You may seek permission from the original copyright holder of Figures 3 and 4 to publish the content specifically under the CC BY 4.0 license.    We recommend that you contact the original copyright holder with the Content Permission Form (http://journals.plos.org/plosone/s/file?id=7c09/content-permission-form.pdf) and the following text:  “I request permission for the open-access journal PLOS ONE to publish XXX under the Creative Commons Attribution License (CCAL) CC BY 4.0 (http://creativecommons.org/licenses/by/4.0/). Please be aware that this license allows unrestricted use and distribution, even commercially, by third parties. Please reply and provide explicit written permission to publish XXX under a CC BY license and complete the attached form.”    Please upload the completed Content Permission Form or other proof of granted permissions as an ""Other"" file with your submission.    In the figure caption of the copyrighted figure, please include the following text: “Reprinted from [ref] under a CC BY license, with permission from [name of publisher], original copyright [original copyright year].”    2.) If you are unable to obtain permission from the original copyright holder to publish these figures under the CC BY 4.0 license or if the copyright holder’s requirements are incompatible with the CC BY 4.0 license, please either i) remove the figure or ii) supply a replacement figure that complies with the CC BY 4.0 license. Please check copyright information on all replacement figures and update the figure caption with source information. If applicable, please specify in the figure caption text when a figure is similar but not identical to the original image and is therefore for illustrative purposes only. | **Figure 3.** We have replaced the images in figure 3 with images published under a CC BY 2.0 licence (the previous image was reproduced under a Microsoft 365 End User Licence Agreement, but we are unsure of the compatibility of this licence with PLoS CC-BY requirements so we changed it). We have included hyperlinks to the source images for licence verification and to support fully attributable reuse.  **Fig 3. Illustration of junction counts in two items of furniture.** Image of chair (A): Just Dining Chairs, Flickr, reproduced under a Creative Commons BY 2.0 licence. Image of sofa (B): Monkeywing, Flickr, reproduced under a Creative Commons BY 2.0 licence.  **Figure 4.** We have replaced the image in figure 4A with an image published under a CC BY 2.0 licence (the previous image was under a CC BY-SA 2.0 licence). The image in figure 4B is published under a CC BY 2.0 licence, so we have not changed it. We have included hyperlinks to the source images for licence verification and to support fully attributable reuse.  **Fig 4. Illustration of a relatively less ornate chair (A) and more ornate sofa (B).** Image of chair: Just Dining Chairs, [Flickr](https://www.flickr.com/photos/156999796@N08/46680444644/), reproduced under a Creative Commons BY 2.0 licence. Image of sofa: Sebastian ter Burg, [Flickr](https://www.flickr.com/photos/ter-burg/14893517887/in/album-72157646604841089/), reproduced under a Creative Commons BY 2.0 licence. (Images also used in Tables 2 and 4.) |
| 4 | 4. Please upload a copy of Figure 15, to which you refer in your text on page 32. If the figure is no longer to be included as part of the submission please remove all reference to it within the text. | This was a typographical error that should have said “Fig 12”. We have corrected this. |
| 5 | 5.Thank you for stating the following in the Competing Interests/Financial Disclosure section:  "PW declares they are a self-employed consultant who provides research, training, and editorial services on [quote snipped to save space here] … Regulations. RH does not believe they have any interests that compete with those of the research described in the manuscript."  We note that one or more of the authors are employed by a commercial company: University of Central Lancashire, Department for Business, Energy, and Industrial Strategy, OPSS,    1.) Please provide an amended Funding Statement declaring this commercial affiliation, as well as a statement regarding the Role of Funders in your study. If the funding organization did not play a role in the study design, data collection and analysis, decision to publish, or preparation of the manuscript and only provided financial support in the form of authors' salaries and/or research materials, please review your statements relating to the author contributions, and ensure you have specifically and accurately indicated the role(s) that these authors had in your study. You can update author roles in the Author Contributions section of the online submission form.    Please also include the following statement within your amended Funding Statement.  “The funder provided support in the form of salaries for authors [insert relevant initials], but did not have any additional role in the study design, data collection and analysis, decision to publish, or preparation of the manuscript. The specific roles of these authors are articulated in the ‘author contributions’ section.”  If your commercial affiliation did play a role in your study, please state and explain this role within your updated Funding Statement.    2.) Please also provide an updated Competing Interests Statement declaring this commercial affiliation along with any other relevant declarations relating to employment, consultancy, patents, products in development, or marketed products, etc.    Within your Competing Interests Statement, please confirm that this commercial affiliation does not alter your adherence to all PLOS ONE policies on sharing data and materials by including the following statement: ""This does not alter our adherence to PLOS ONE policies on sharing data and materials.” (as detailed online in our guide for authors <http://journals.plos.org/plosone/s/competing-interests>) . If this adherence statement is not accurate and there are restrictions on sharing of data and/or materials, please state these. Please note that we cannot proceed with consideration of your article until this information has been declared.    Please include both an updated Funding Statement and Competing Interests Statement in your cover letter. We will change the online submission form on your behalf. | (1) To clarify, neither University of Central Lancashire (UCLAN), nor the Department for Business, Energy, and Industrial Strategy, (BEIS) nor the Office for Product Safety and Standards (OPSS) are commercial entities.   - UCLAN is a university - BEIS is a UK government agency - OPSS is a part of BEIS   The employment status of each author in relation to their respective employers, and the role of the funder (BEIS and OPSS) and the contractee (UCLAN), are described in the declarations of interest and the funding statement as per comment 2 above.  For the BEIS employees (TK and DN) we have added a clarifying statement to their declarations of interest, stating that they worked “in the science division (Research, Evidence and Dissemination team) within OPSS” and their “role in this context was to provide scientific and technical advice to policy makers, and was not themselves a decision-maker or policy lead.”  We believe our funding statement (see comment 2 above and the cover letter) is comprehensive and correct in its description of the role of the funder (employing two of the contributors as technical advisers plus applying an internal clearance process before approval for publication) and the affiliations of all authors.  (2) We believe our competing interests statements are comprehensive and clearly describe employment status, funding sources, and other interests of each author as appropriate. We are not sure what specifically should be changed in order to be compliant with PLoS ONE policies - more guidance on this would be welcome.  As requested, we have added the statement “These interests do not alter our adherence to PLOS ONE policies on sharing data and materials” to our declaration of interests statement (see cover letter).  If the Editor could provide further guidance on what would need to be changed about the funding statement and declarations of interest to comply with PLoS policies, we would be more than happy to make any further changes as needed.  Both the Funding Statement and Competing Interests statement are in our cover letter. |
| 6 | 6. We note that you have stated that you will provide repository information for your data at acceptance. Should your manuscript be accepted for publication, we will hold it until you provide the relevant accession numbers or DOIs necessary to access your data. If you wish to make changes to your Data Availability statement, please describe these changes in your cover letter and we will update your Data Availability statement to reflect the information you provide. | Anticipating publication, we have uploaded all supporting material and made it publicly accessible on Zenodo, here: <https://doi.org/10.5281/zenodo.8351890>  We have included in the cover letter the following data availability statement, as requested: “All data for this study are fully available without restriction, from<https://doi.org/10.5281/zenodo.8351890>” |
| 7 | 7. We note you have included a table to which you do not refer in the text of your manuscript. Please ensure that you refer to Tables 3 and 6 in your text; if accepted, production will need this reference to link the reader to the Table. | Thank you for noticing this. All tables are now referenced in the main text. |
| 8 | 8. We notice that your supplementary "INDEX OF SUPPLEMENTAL MATERIALS" are included in the manuscript file. Please remove them and upload them with the file type 'Supporting Information'. Please ensure that each Supporting Information file has a legend listed in the manuscript after the references list. | We have created a “Supporting information” file and added legends for the supporting information after the references list, as requested. This includes a link to the data on Zenodo. |
| 9 | 9. Please review your reference list to ensure that it is complete and correct. If you have cited papers that have been retracted, please include the rationale for doing so in the manuscript text, or remove these references and replace them with relevant current references. Any changes to the reference list should be mentioned in the rebuttal letter that accompanies your revised manuscript. If you need to cite a retracted article, indicate the article’s retracted status in the References list and also include a citation and full reference for the retraction notice. | We confirm that our reference list is complete and includes no retracted articles. |
| Comments from Reviewer 1 | | |
| 10 | Overall evaluation   - The topic of furniture as exposure source of hazardous chemicals is of contemporary interest for the research community, for policy makers and the related industries as well as for the public. - The topic is also in the scope of the journal. - The paper can in my opinion be accepted after minor revisions. | We thank the reviewer for their helpful comments and provide responses below. |
| 11 | You lump all chemical flame retardants together which is an oversimplification. Please mention that there are e.g. halogenated flame retardants which are considered particular problematic. Possibly mention the review article of Shaw et al. 2010.  Shaw SD, Blum A, Weber R, Kannan K, Rich D, Lucas D, Koshland CP, Dobraca D, Hanson S, Birnbaum LS. (2010) Halogenated Flame Retardants: Do the Fire Safety Benefits Justify the Risks? Rev. Environ. Health 25(4), 261-305. | We agree that different subclasses of CFR pose different environmental and human exposure issues, and that halogenated CFRs are historically viewed as the most problematic. However, our manuscript concerns the potential for exposure to CFRs as a class of chemical in general, so our “lumping” is deliberate and intrinsic to the study goals. Adding discussion of the challenges of different classes of CFR would lengthen the introduction and potentially be confusing in an already lengthy, complex methods paper that, in our opinion, is not intended to be a place for discussing the broader issues around CFR use. |
| 12 | Please mention that almost all other countries do not have this flame retardant standard which require that the bulk polymer in furniture is flame retarded and that in particular UK and US had this stringent standard. Please mention that US/California modified their flammability standard for furniture which does not require to use flame retardants for the bulk polymer/PUR now. Please reference to Charbonnet et al. 2020 and possibly others.  Charbonnet, J.A., Weber, R. and Blum, A., 2020. Flammability standards for furniture, building insulation and electronics: Benefit and risk. Emerging Contaminants, 6, pp.432-441. | We have added the reference to Charbonnet et al. and a sentence to the end of paragraph 1 of the introduction, stating: “In comparison, regulations in the European Union and the United States specify limits to flammability in a way that does not drive the addition of flame retardants to furniture fillings.” |
| 13 | Please mention the situation of other European countries (also almost all other countries): Most of them do not use flame retardants in PUR foam in furniture. I do not think that they experience unacceptable fire risks. Please mention and discuss. If possible please compare fire death or fire frequency in at least some European countries which do not require flame retardants in bulk materials in furniture with UK. | We acknowledge this is important context. However, due to lack of like-for-like data, a balanced and rigorous analysis of relative fire frequency and fire deaths between the UK and EU countries is very challenging to undertake, particularly the fraction that is specifically attributable to furniture. We view this as being beyond the scope of this manuscript, which is already lengthy and concerns a method for balancing fire risk against CFR exposure rather than being a place for discussion of the relative merits of or justification for FR use; nonetheless, we are very much aware of these issues and there is a substantial annex in the report relating to this publication (Annex 1, [here](https://www.gov.uk/government/publications/fire-risks-of-upholstered-products)).  To at least signpost for the reader where they can access this contextual information, we have edited the following sentence at the end of the Objectives section: “This paper presents for a scientific audience some of the research described in the UK Office for Product Safety and Standards report Fire risks of upholstered products. This report includes extensive context on CFR technologies and provides statistics on international incidence of fires and fire deaths (18).” |
| 14 | Line 100: “The most widely used CFRs in domestic furniture include decabromodiphenyl ethane (DBDPE) “. Please mention that this is today and that in the past decaBDE was mainly used. Also please mention that PBDEs are listed as POPs in the Stockholm Convention for global phase out. | Our study is motivated by contemporary use of CFRs. We have added “currently” to line 75 to make it clear that we are talking about the present situation. However, in our opinion the history of use of CFRs has no direct bearing on our methods paper (that is already lengthy and complex) and has also been well-documented elsewhere. |
| 15 | Line 107: “DBDPE to Deca-BDE suggests that it too will be withdrawn at some point in the near future: it is currently under assessment as PBT (ECHA, 2018).” Please mention here the new proposed strategy of ECHA for flame retardants with suggestion to phase out aromatic brominated flame retardants as a group. | The proposed ECHA strategy we believe the reviewer is referring to ([here](https://echa.europa.eu/documents/10162/2082415/flame_retardants_strategy_en.pdf/9dd56b7e-4b62-e31b-712f-16cc51d0e724?t=1679045593845)) is still at a very preliminary stage and, as we understand it, also includes groupings for organophosphorous CFRs. We are not confident that this information provides appropriate context for justifying the conduct of our study. |
| 16 | Line 117ff “Meanwhile, pressures such as reducing the presence of toxic substances in our environment, the need to recycle products such as furniture at their end-of-life, and the “unsuitability of furniture containing CFRs for alternative end-of-life processes, such as landfill or material reclamation, are much higher priorities than they were in the 1980s.” Not clear – please rephrase. | We have amended the sentence for clarity. |
| 17 | Line 387ff: “We do, however, know that none of the reviews in our document set that addressed human exposure to flame retardants discussed in detail how specific items of furniture contribute to a person’s exposure to CFRs.”  Abdallah & Harrad (2018) have conducted studies which showed that HBCD in furniture textiles result in relevant exposure by skin contact. Please mention this and reference to Abdallah, M.A.E. and Harrad, S., 2018. Dermal contact with furniture fabrics is a significant pathway of human exposure to brominated flame retardants. Environment international, 118, pp.26-33.  Also Pawar, G., Abdallah, M.A.E., de Sáa, E.V. and Harrad, S., 2017. Dermal bioaccessibility of flame retardants from indoor dust and the influence of topically applied cosmetics. Journal of exposure science & environmental epidemiology, 27(1), pp.100-105.  You mention the first paper in the Table 3 but suggest to mention it also in the text. | The studies referenced by the reviewer do provide evidence of how textile contact can lead to CFR exposure, but they do not allow relative exposure to CFRs via different routes to be calculated. We are therefore unsure of the relevance of this citation to the claims being made in this section of the manuscript. |
| 18 | Table 3: (Abou-Elwafa Abdallah and Harrad, 2022). Think this is (Abdallah and Harrad, 2022). Also in his researchgate he abbreviate to Mohammed A. Abdallah) | We would ask the handling editor to advise on this. We appreciate that the author in question has a preferred name, but the metadata for the citation in question uses the name as we have referenced. |
| 19 | Table 5: Baby mattress has lower score (3.4) compare to Baby products with seat (3.6) and Bassinet (4.0). However mattress has likely the by far longest contact time. Possibly modify? | Baby mattresses scored high in exposure domains where baby products with a seat tended to score low, so they ended up with similar exposure ratings overall (see exposure model scores tab of SM15). We cannot modify the scores as this would be fabrication of data. We could weight duration more heavily in the overall rating but, as we explain in our methods section, we do not believe such a weighting can be justified given the data we have available to us. |
| 20 | Line 566: “moved around, so were judged to be more likely to come into contact with smokers materials.” While smokers materials are certainly relevant, suggest also to mention candles having high use frequency. | We have edited the sentence to state “smokers materials or candles” |
| Comments from Reviewer 2 | | |
| 21 | The manuscript is exceptionally well-written, providing valuable insights into future fire risk assessment and CFR exposure modeling concerning furniture. The comprehensive explanation and presentation of limited information and empirical data regarding the flammability of fabric and foam when exposed to small open flames and materials used by smokers are noteworthy. There are no discernible flaws in this publication, and it is ready for publication as is. | Thank you for this very generous comment. We are making framed copies for our offices. |
